# Effect of mobile application user interface improvements on minimum expected home visit coverage by community health workers in Mali: a randomised controlled trial

**DOI:** 10.1136/bmjgh-2021-007205

**Published:** 2021-11-21

**Authors:** Jane E Yang, Diego Lassala, Jenny X Liu, Caroline Whidden, Isaac Holeman, Youssouf Keita, Yasamba Djiguiba, Sory Ibrahima N’Diaye, Fatou Fall, Kassoum Kayentao, Ari D Johnson

**Affiliations:** 1 Muso, San Francisco, California, USA; 2 Muso, Bamako, Mali; 3 Department of Social and Behavioral Sciences, University of California San Francisco, San Francisco, California, USA; 4 Medic, San Francisco, California, USA; 5 Department of Human Centered Design & Engineering, University of Washington, Seattle, Washington, USA; 6 Department of Global Health, University of Washington, Seattle, Washington, USA; 7 Medic, Dakar, Senegal; 8 Malaria Research & Training Centre, University of Sciences Techniques and Technologies, Bamako, Mali; 9 Department of Medicine, Institute for Global Health Sciences, University of California San Francisco, San Francisco, CA, USA

**Keywords:** health services research, public health, randomised control trial, prevention strategies, other diagnostic or tool

## Abstract

**Background:**

Proactive community case management (ProCCM) has shown promise to advance goals of universal health coverage (UHC). ProCCM community health workers (CHWs) face operational challenges when pursuing their goal of visiting every household in their service area at least twice monthly to proactively find sick patients. We developed a software extension (UHC Mode) to an existing CHW mobile application featuring user interface design improvements to support CHWs in planning daily home visits. We evaluated the effect of UHC Mode on minimum expected home visit coverage.

**Methods:**

We conducted a parallel-group, two-arm randomised controlled trial of ProCCM CHWs in two separate regions in Mali. CHWs were randomly assigned to UHC Mode or the standard mobile application (control) with a 1:1 allocation. Randomisation was stratified by health catchment area. CHWs and other programme personnel were not masked to arm allocation. CHWs used their assigned intervention for 4 months. Using a difference-in-differences analysis, we estimated the mean change in minimum expected home visit coverage from preintervention to postintervention between arms.

**Results:**

Enrolment occurred in January 2019. Of 199 eligible CHWs randomised to the intervention or control arm, 196 were enrolled and 195 were included in the analysis. Households whose CHW used UHC Mode had 2.41 times higher odds of minimum expected home visit coverage compared with households whose CHW used the control (95% CI 1.68 to 3.47; p<0.0005). Minimum expected home visit coverage in the UHC Mode arm increased 13.6 percentage points (95% CI 8.1 to 19.0) compared with the control arm.

**Conclusion:**

Our findings suggest UHC Mode is an effective tool that can improve home visit coverage and promote progress towards UHC when implemented in the ProCCM context. User interface design of health information systems that supports health workers’ daily practices and meets their requirements can have a positive impact on health worker performance and home visit coverage.

**Trial registration number:**

NCT04106921.

**Key questions**

**What is already known?**

- There are few published studies that have evaluated the effect of digital user interface design interventions on health worker performance or health service delivery in low-income and middle-income country settings.
- Most studies of user interface design have methodological limitations and focus on qualitative surveys of the acceptability and usability of health information systems.
- This research gap is notable given existing strategies to improve service coverage and access to care through community health workers (CHWs), and considering CHWs may have more limited access to technical literacy training opportunities than is the norm among physicians and nurses.

**What are the new findings?**

- This randomised controlled trial is one of the first to evaluate the effect of digital user interface design improvements on CHW performance, specifically minimum expected home visit coverage.
- Use of universal health coverage (UHC) Mode, a software extension to an existing mobile application for CHWs, resulted in an increase in minimum expected home visit coverage.
- Across two study areas, results were similarly positive, though the separate analysis for the rural health catchment area was limited by its small sample size.

**Key questions**

**What do the new findings imply?**

- As countries pursue their 2030 UHC goals, digitally supported CHWs can advance these goals at the local level.
- UHC Mode should be considered an effective tool that can improve minimum expected home visit coverage and promote progress towards UHC when implemented in the proactive community case management context.
- User interface design of health information systems that supports health workers in their daily practice and meets their requirements can have a positive impact on health worker performance and home visit coverage.

## Introduction

Achieving universal health coverage (UHC), a critical sustainable development goal and a precursor to meeting other health-related sustainable development goals such as ending preventable child and maternal deaths, requires monitoring population coverage of essential health services and attaining high rates of coverage and access.^1^ To make progress towards UHC in sub-Saharan Africa and improve child health and survival, many governments have implemented a strategy of community-based service delivery known as integrated community case management (iCCM) via community health worker (CHW) programmes at varying levels of scale.^2^ This strategy consists of the delivery of essential health services by trained health workers who live in the communities they serve. Although a range of studies show that CHWs can effectively deliver services,^3–5^ many low-income and middle-income country (LMIC) settings have observed low coverage of home visits and other health services.^6–10^

Proactive CCM (ProCCM) is an alternative iCCM model that aims to increase coverage, speed and quality of health service delivery and improve maternal and child health outcomes. ProCCM CHWs integrate into existing health systems, receive monthly dedicated supervision, conduct active case-finding home visits to all households in their service area, provide doorstep care free-of-charge to patients, and evacuate patients with danger signs to redesigned government primary care clinics. After promising findings from recent observational studies,^11^ ProCCM and the causal effects of proactive case detection on access to care and child mortality are being evaluated in rural Mali.^12^ CHW workflow for service delivery, the effect of home visits on access to care, and ways to optimally support CHW performance remain research priorities in the community health field.^13–15^

Digital technologies such as mobile applications are recommended as a best practice for high-functioning CHW programmes to improve the speed, quality and equity of CHW services via more timely and usable data.^16^ Digital health interventions may increase adherence to case management guidelines,^17 18^ increase timeliness of care,^19^ and improve the effectiveness of CHW supervision.^15^ Yet they also face complex implementation challenges, including the need for better alignment with health worker routines and interoperability with local infrastructure.^20^ Poor accessibility of user interfaces may limit the benefits of health information systems and has been cited as a source of medical and user errors and health worker burnout.^21–23^ There are still very few studies of the effects of improved user interface design on digitally supported care in LMICs. This gap is notable given existing strategies to improve service coverage and access to care through CHWs, and considering CHWs may have more limited access to technical literacy training opportunities than is the norm among physicians and nurses.^24^

In a CHW programme in Mali, ProCCM CHWs are often each responsible for 100–200 households depending on the setting (rural or urban) and population density. Given their context-specific knowledge of their communities and responsibilities for triaging a wide array of tasks, including urgent patient evacuations, ProCCM CHWs maintain complete autonomy in determining their daily workflows (eg, the sequence of visiting households in the service area). Still, they face unique operational challenges as they pursue meeting the programmatic goal of visiting every household in their service area at least twice monthly, including lack of functionality in their existing mobile application to monitor their progress and organise their workflow. We developed a software extension (UHC Mode) to address these challenges by providing CHWs with real-time data to support CHWs in planning their daily home visits. This study’s primary objective was to evaluate the effect of UHC Mode on minimum expected home visit coverage.

## Methods

### Study design and participants

**Figure 1:**
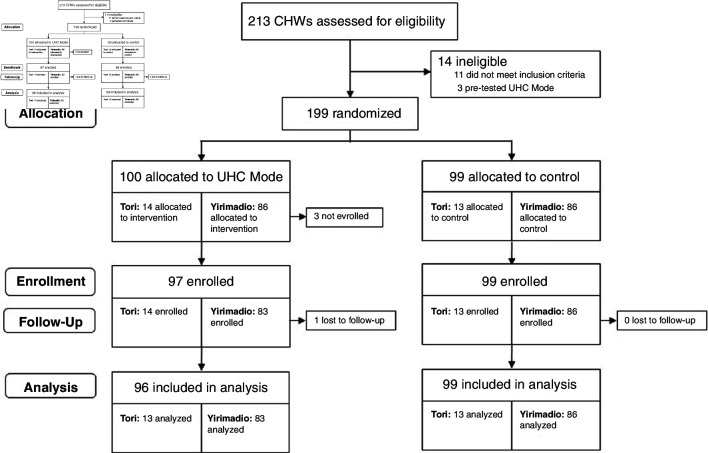
Trial profile. CHWs, community health workers; UHC, universal health coverage.

We conducted a parallel-group, two-arm randomised controlled trial in the community setting in two health catchment areas in separate regions in Mali, from August 2018 to July 2019 (see figure 1 for Consolidated Standards of Reporting Trials diagram).

CHWs in Tori and Yirimadio providing health services based on the ProCCM model were eligible. Although this study took place in a larger context of ProCCM care delivery, a full description of which is published elsewhere,^11 12^ it is unrelated to the separate ProCCM trial.^12^ Tori, a rural area in the Mopti region in central Mali, had a population of 29 029 in 2019, while Yirimadio, a periurban area in Bamako, the nation’s capital, had an estimated population of 176 089. Both areas were each served by one public sector primary health centre at the time of the trial.

Three CHWs delivering ProCCM in Yirimadio were randomly selected to pilot test UHC Mode before its launch. CHWs who did not provide written informed consent, or had to drop out of the study before intervention launch, or were involved in pilot testing, were excluded from the trial.

Although additional secondary outcomes were originally planned, these were dropped due to operational constraints and unforeseen challenges in study implementation (described in the Data preparation section of online supplemental materials).

10.1136/bmjgh-2021-007205.supp1 Supplementary data

### Randomisation and masking

Eligible CHWs not involved in pilot testing were randomly assigned to the control or UHC Mode arm with a 1:1 allocation using a computer-generated random number. Randomisation was stratified by health catchment area, and performed by a research team member who was not involved in study recruitment. Due to operational constraints, randomisation was done before study recruitment. The nature of UHC Mode precluded CHW participants, CHW supervisors and other study personnel from being masked to arm allocation. To avoid contamination, two training sessions were held before intervention launch: one for all eligible CHWs to discuss standard definitions of households and home visit protocols, and a second for CHWs randomised to UHC Mode, to provide an overview of its features, and discourage discussions about UHC Mode with CHWs in the control arm.

### Procedures and study period

During the study period, eligible CHWs followed the standard ProCCM protocol: conduct home visits for a minimum of 2 hours per day, 6 days per week, in order to visit each household in their service area with the minimum expected frequency (at least twice monthly).^11 12^ During their home visits, CHWs reinforced the importance of rapid care-seeking and encouraged community members to call their mobile telephone number or visit them right away if their child became symptomatic. CHWs were expected to be on call to provide care outside of their regular home visiting hours.^12^

CHWs were also expected to register and maintain information on all households and patients in their service area and to document each home visit and sick patient evaluation using a CHW application preinstalled on Android phones. CHWs maintained autonomy and flexibility in planning their daily home visit workflows, including what order they visited households. Preintervention (baseline) was defined as August 2018 to March 2019. In the latter half of preintervention (December 2018–March 2019), a CHW Supervision Dashboard was implemented in both arms to enable dedicated CHW supervisors to monitor CHW performance indicators related to timeliness and quantity of monthly home visits and quality of care.^15^ For 4 months postintervention (April–July 2019), eligible CHWs used their randomised intervention: the standard CHW application (control) or the CHW application with UHC Mode (intervention). UHC Mode was deployed in Yirimadio on 8 March 2019 and in Tori on 13 March 2019.

### The intervention and control

**Figure 2:**
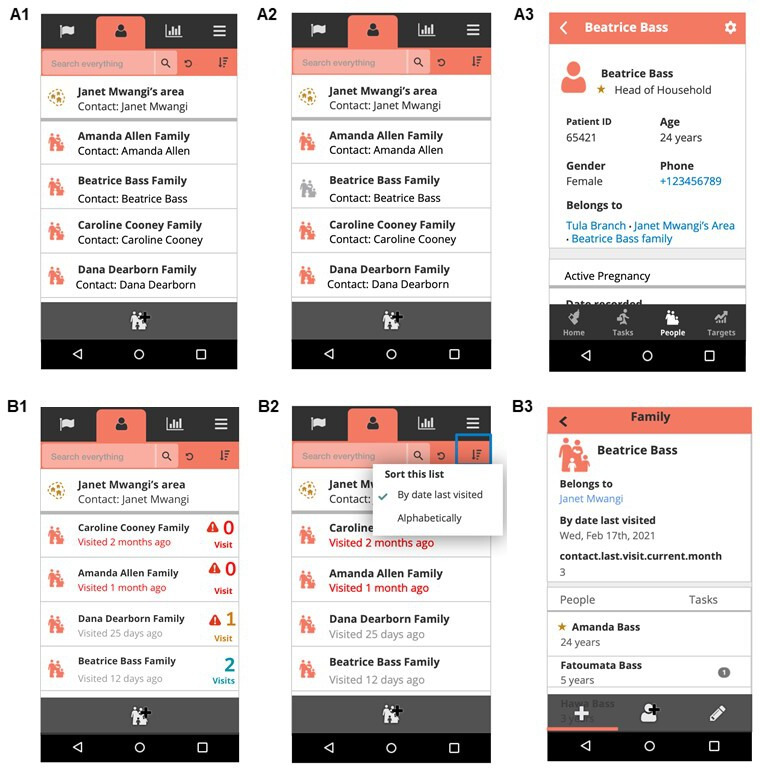
Display of information on the household list in the standard CHW application (A1–A2) versus UHC Mode (B1–B2) and on individual household profiles in the standard application (A3) versus UHC Mode (B3). A1 shows the household list in the standard CHW application. A2 shows the household list in the standard CHW application, with default alphabetical ordering. B1 shows the household list in UHC Mode, which displays the time elapsed in days since the last home visit (colour coded red if the date of last visit was 30 or more days ago, and black if the date of last visit was fewer than 30 days ago). Red exclamation point icons emphasise households with fewer than two visits in the month, and a modified traffic light colour scheme (red, orange, light blue) show households receiving zero, one, or two or more visits in the month. B2 shows the household list in UHC Mode, with default chronological ordering by date of last home visit. A3 shows an individual household profile in the standard CHW application. B3 shows an individual household profile in UHC Mode, with information about the date of last visit and the monthly home visit count. CHW, community health worker; UHC, universal health coverage.

UHC Mode is an add-on (software extension) to the CHW application consisting of user interface design improvements to support CHWs to achieve their minimum expected home visit coverage of each household in their service area each month. It features four main design elements: First, each household’s date of last visit is displayed on individual household profiles, which show households’ primary contact information and registered members. This information is displayed on the household list as the time elapsed (in days or months) since the last home visit and colour coded red (if the date of last visit was 30 or more days ago) or black (if the date of last visit was fewer than 30 days ago). Second, red exclamation point icons appear on the household list to emphasise households with fewer than two visits in the month. Third, a modified traffic light colour scheme (red, orange, light blue) shows households receiving zero, one or two or more visits during the month. Finally, the default ordering of the household list was changed from alphabetical by name to chronological by least recent visit date (figure 2).

UHC Mode was the result of an iterative, human-centred design process,^20^ which involved direct observations of CHWs planning their daily home visits, focus groups with CHWs and CHW supervisors to elicit feedback on design features to promote higher minimum expected home visit coverage, and months of iterative pilot testing to incorporate CHW feedback into prototype development. The initial prototype focused on redesigning the patient lists feature, which CHWs had identified in focus groups as a bottleneck to managing their daily home visit workflows. Prototype development was informed by key user experience principles discussed in design literature^25^: visibility, feedback and positive reinforcement. The aim was to increase the visibility of minimum expected home visit coverage information, to provide users with clear feedback whenever their actions had contributed to the goal of minimum expected home visit coverage, and to provide feedback in a way that positively reinforced practices conducive to high coverage rates.

The control was the standard CHW application used by all eligible CHWs before the study, which was built with the open-source Community Health Toolkit.^26^ Key features include job aids for each care protocol, a task list, a report tab summarising CHW performance related to predefined targets, and a patient list organised by household, with access to patients’ longitudinal records. CHWs can organise their daily workflow using the task management, targets and patient list features, and administer clinical care protocols with job aids at the point of care. The application is designed to work while offline and to sync data to a central server when connectivity is available.

Important preconditions to UHC Mode’s successful implementation identified during pilot testing were incorporated into the CHW application by intervention launch and available to CHWs in both arms. These included faster application performance, and new functionality (household ‘muting’ forms) to enable CHWs to deactivate (and reactivate) households that moved from the CHW service area (temporarily or permanently), declined CHW services, or no longer required CHW services for other reasons.

### Data collection

Data on home visits were collected using the CHW application preintervention and via the assigned intervention postintervention, and extracted more than 3 months after the study period. We counted a maximum of one visit per unique household per day, to reduce the potential for report duplication. If a CHW recorded more than one visit for the same household on the same day, then only one of those visits was counted. Observations were included in the analysis if households were registered and visits were conducted within the study period, and households were considered active (household still present based on household muting form information) for at least 2 days in the month. Data were prepared and analysed using Stata V.15 (Stata). Additional information on data sources and preparation are given in online supplemental appendix 1.

### Outcomes

We used a binary primary outcome variable, whether an individual household in the CHW service area had minimum expected home visit coverage (at least two home visits by the CHW) in the month.

### Statistical analysis

The primary outcome measure was the mean change in minimum expected home visit coverage (defined as an individual household’s receipt of at least two visits in the month) from preintervention to postintervention between arms. We also calculated the service area coverage of minimum expected home visits (the percentage of households visited at least twice in a month) by arm and the unadjusted difference from preintervention to postintervention between arms.

Study sample size was primarily determined by operational constraints; all available ProCCM CHWs in Tori and Yirimadio were eligible for the study.

We first performed a descriptive analysis to assess comparability between arms in terms of CHWs’ sociodemographic characteristics, baseline service area coverage, actual household load and the wealth quintile distribution among households in the service area. Calculation of actual household load (the number of active households in the CHW service area) was enabled by the household muting functionality incorporated into the CHW application in both arms by intervention launch. To allow sufficient time for CHWs to implement the functionality, we used the number of active households in the final month of the postintervention period and applied it to all earlier months, assuming this was constant during the study period. We next calculated the unadjusted difference in CHW service area coverage from preintervention to postintervention between arms. Service area coverage was graphed to compare trends by arm and month, and preintervention versus postintervention. Comparisons were done with health catchment areas combined and stratified given that baseline service area coverage varied by health catchment area.

To analyse the primary outcome measure and estimate UHC Mode’s effect on CHW coverage of a given household in the service area with minimum expected home visits, we conducted a difference-in-differences analysis using a logit model for panel data with CHW-level random effects:

$$Y_{hiam}=\alpha +\beta_{1}T_{i}+\beta_{2}P_{m}+\beta_{3}T_{i}*P_{m}+\beta_{4}Z_{a}+e_{hiam}$$

where *Y* is the log odds of coverage of minimum expected home visits observed for household *h* by CHW *i* in health catchment area *a* in month *m; T_i_* is an indicator variable for a CHW’s arm allocation; *P_m_* is an indicator variable for the postintervention period; Z*_a_* is an indicator variable for health catchment area; and *e_him_* is the idiosyncratic error term. The *β_3_* coefficient on the interaction term, T_i_*P_m_, estimates the difference-in-differences in the log odds of minimum expected home visit coverage, comparing the mean change (from preintervention to postintervention) in the log odds of minimum expected home visit coverage in households whose CHW used UHC Mode versus the mean change in households whose CHW used the control. We used the margins command in Stata to estimate the absolute change in the predicted probabilities of minimum expected home visit coverage in the UHC Mode versus the control arm. SEs were clustered by CHW, the unit of randomisation. Sensitivity analyses were performed to check the robustness of our regression model to controlling for: month fixed effects (to account for any systematic variations over time); alternative definitions of the preintervention period (ie, 4 months with the CHW Supervision Dashboard and dropping the transition month when UHC Mode launched); and including zero home visit counts for households indicated by CHWs to be temporarily or permanently inactive (to account for bias from potential under-reporting in the event that CHWs who used UHC Mode were more likely than CHWs who used the control to report inactive households).

In additional exploratory analyses, we assessed differences in intervention effects by health catchment area (Tori vs Yirimadio), levels of baseline service area coverage, and levels of actual household load. We also performed a subgroup analysis to evaluate whether UHC Mode improved minimum expected home visit coverage of households in the poorest wealth quintile compared with richer households and potentially improved equity in service delivery. This subgroup analysis was limited to Tori because household wealth information was unavailable for Yirimadio, and programme records showed that households in the poorest wealth quintile in Tori had significantly lower odds of minimum expected home visit coverage compared with households in higher wealth quintiles.

For exploratory analyses, we repeated the overall main effects analysis, adding a three-way interaction term including one of the following: a dichotomous variable for health catchment area; a categorical indicator variable for baseline service area coverage quartiles; a categorical indicator variable for actual household load quintiles; or a categorical indicator variable for household wealth quintiles (Tori only). The percentile variables for baseline service area coverage and actual household load were calculated separately by health catchment area. We obtained household wealth quintile information for Tori by conducting a principal component analysis of household survey data on household assets,^12^ adapted from the Demographic and Health Survey.

### Patient and public involvement

Design of the intervention was informed by engaging with, and soliciting input from, CHWs and CHW supervisors. Patients and the public were not directly involved in the study design, conduct, reporting or dissemination plans of this research. The two study sites are longstanding operational research sites, where Muso and its partners have worked in close partnership with local communities for years. Muso began operational research to improve access to care, maternal health, and child survival in Yirimadio in 2005, and in Tori in 2016. In each area, Muso works closely with local Ministry of Health officials and community members via the Associations de Santé Communitaire, Community Health Associations. These community partners have authorised Muso to conduct operational research related to ProCCM, and actively engage as partners in this research.

## Results

Of 213 CHWs assessed for eligibility, 199 were eligible and randomised to receive UHC Mode (n=100) or control (n=99) in early January 2019 (figure 1). Of eligible and randomised CHWs, 196 (97 UHC Mode, 99 control) were recruited in CHW meetings held 2 January 2019–16 January 2019 (Yirimadio) and 25 January 2019–26 January 2019 (Tori). Three eligible CHWs in Yirimadio randomised to UHC Mode were unable to attend the CHW meetings when study enrolment occurred due to health reasons. There were 195 CHWs (96 UHC Mode, 99 control) included in this analysis; one CHW in Tori randomised to UHC Mode dropped out of the study before intervention launch due to health reasons.

CHWs served a total of 45 873 unique households (4288 in Tori, 41 585 in Yirimadio) during the study period. CHW sociodemographic and service provision characteristics at baseline are shown in tables 1 and 2.

**Table 1:** CHW baseline characteristics

	UHC mode (n=96)	Control (n=99)	P value
Age, years	32.0 (8.7)	33.0 (9.6)	0.46
No of children	2.9 (2.1)	3.2 (2.3)	0.48
Household size	6.4 (3.0)	6.2 (2.6)	0.60
No of spoken languages	1.3 (0.8)	1.2 (0.6)	0.36
Sex			
Male	10 (10%)	6 (6%)	0.27
Female	86 (90%)	93 (94%)	
Religion			
Muslim	92 (96%)	95 (96%)	0.26
Catholic	4 (4%)	2 (2%)	
Other	0 (0%)	2 (2%)	
Marital status			
Single	15 (16%)	15 (15%)	0.74
Married/free union	76 (79%)	81 (82%)	
Divorced/widow/separated	5 (5%)	3 (3%)	
Language			
Bambara	83 (86%)	88 (89%)	0.56
French	7 (7%)	8 (8%)	
Sorai	6 (6%)	3 (3%)	
Has children			
Yes	84 (88%)	87 (88%)	0.94
No	12 (12%)	12 (12%)	
Went to school			
Yes	95 (99%)	97 (98%)	0.58
No	1 (1%)	2 (2%)	

Data are n (%) or mean (SD). Statistical tests were performed using a two-sided t-test of means for continuous variables and Pearson’s χ^2^ test for categorical variables.

CHW, community health worker; UHC, universal health coverage.

**Table 2:** Baseline service provision characteristics

Cumulative household registrations	UHC mode (n=96)	Control (n=99)	P value
Overall	230.0 (83.3)	240.4 (131.7)	0.51
Yirimadio	239.8 (83.7)	252.1 (136.6)	0.48
Tori	167.0 (45.4)	162.8 (44.7)	0.82
Actual household load			
Overall	192.1 (65.2)	206.1 (103.9)	0.26
Yirimadio	196.8 (67.0)	212.7 (108.8)	0.25
Tori	162.5 (43.2)	162.5 (44.6)	1.0
Service area coverage			
Overall	46.6 (21.4)	44.7 (21.3)	0.54
Yirimadio	44.0 (20.9)	43.2 (21.1)	0.80
Tori	63.2 (17.3)	54.7 (20.3)	0.26

Household wealth quintile distribution	UHC Mode (n=1719)	Control (n=1782)	P value
Quintile 1 (lowest)	262 (15%)	263 (15%)	0.64
Quintile 2	270 (16%)	306 (17%)	
Quintile 3	342 (20%)	372 (21%)	
Quintile 4	414 (24%)	405 (23%)	
Quintile 5 (highest)	431 (25%)	436 (25%)	

Data are mean (SD) or n (%).

CHW, community health worker; UHC, universal health coverage.

The mean cumulative number of households registered, actual household load, and baseline service area coverage did not differ between arms, but did differ by health catchment area (table 2). In the UHC Mode arm, mean baseline service area coverage across CHWs was 44.0% (SD=20.9%) in Yirimadio compared with 63.2% (SD=17.3%) in Tori; the mean number of cumulative households registered by CHWs was 239.8 (SD=83.7) in Yirimadio compared with 167.0 (SD=45.4) in Tori; and the mean actual household load was 196.8 (SD=67.0) in Yirimadio compared with 162.5 (SD=43.2) in Tori.

The unadjusted difference in CHW service area coverage in the UHC Mode arm from preintervention to postintervention was 15.5 (95% CI 9.8 to 21.2; p<0.0005) percentage points higher than in the control arm (table 3).

**Table 3:** Percentage of households visited at least twice in a month by arm and preintervention and postintervention periods, and differences in differences from preintervention to postintervention between arms, with health catchment areas combined and stratified

	Preintervention* (August 2018–March 2019)		Postintervention* (Apri–July 2019)		DID % point change*		DID OR†	
	UHC mode (n=68 154)	Control (n=67 364)	UHC mode (n=52 562)	Control (n=42 293)	DID (95% CI)	P value	OR (95% CI)	P value
Households visited at least twice in a month								
Combined	43.1 (38.7, 47.7)	39.5 (34.4, 44.8)	70.9 (64.7, 76.4)	51.8 (45.2, 58.3)	15.5 (9.8 to 21.2)	<0.0005	2.41 (1.68 to 3.47)	<0.0005
Yirimadio	n=57 533	n=58 480	n=44 997	n=36 344	16.1 (10.2 to 22.0)	<0.0005	2.40 (1.66 to 3.49)	<0.0005
	40.9 (36.3–45.6)	38.1 (32.7, 43.7)	68.6 (61.9, 74.5)	49.6 (42.7, 56.6)				
Tori	n=10 621	n=8884	n=7565	n=5949	10.0 (-5.9 to 25.9)	0.23	3.31 (0.80 to 13.67)	0.098
	61.5 (49.1, 72.6)	52.6 (40.0, 64.8)	89.3 (81.7, 94.0)	70.4 (54.4, 82.5)				

All estimates are adjusted for clustering at the CHW level.

*Preintervention and postintervention service area coverage and DID percentage point changes are unadjusted for covariates.

†Estimates for a random effects panel regression, with a treatment indicator that takes value 0 for the control arm and value 1 for the intervention arm. Combined estimates control for health catchment area.

CHW, community health worker; DID, difference-in-differences; UHC, universal health coverage.

**Figure 3:**
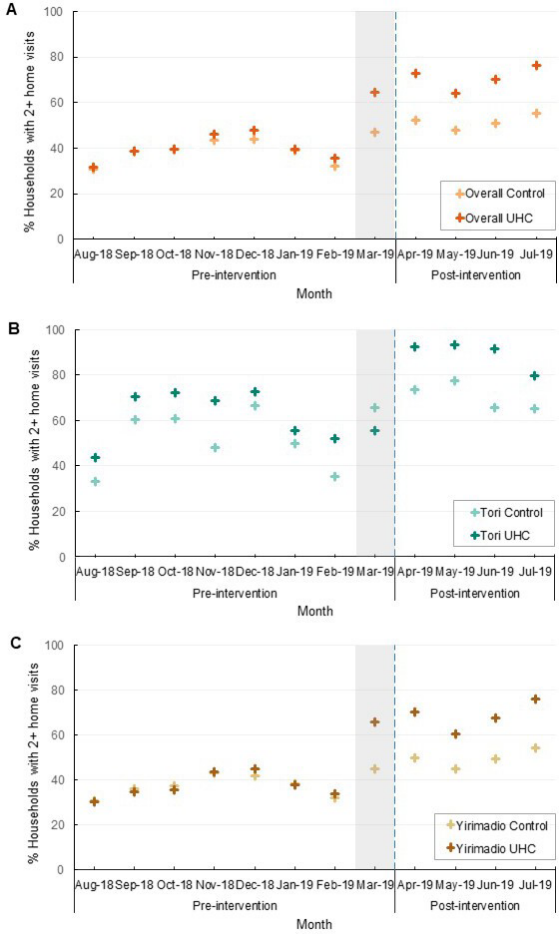
Monthly and preintervention versus postintervention CHW service area coverage of minimum expected home visits (percentage of households visited at least twice in a month), by arm, with health catchment areas combined (A) and stratified (B–C). Vertical blue dotted lines show the formal start of the postintervention period. Gray-shaded areas show the roll-out of UHC Mode in March 2019. CHWs, community health workers; UHC, universal health coverage.

In the UHC Mode arm, baseline service area coverage was 43%, increasing to 71% postintervention. In the control arm, baseline service area coverage was 40%, increasing to 52% postintervention (table 3, figure 3).

Households whose CHW used UHC Mode had 2.41 times higher odds of minimum expected home visit coverage from preintervention to postintervention compared with households whose CHWs used the control (95% CI 1.68 to 3.47; p<0.0005) (table 3). On average, minimum expected home visit coverage in the UHC Mode arm increased 13.6 percentage points (95% CI 8.1 to 19.0) compared with the control arm. The odds of minimum expected home visit coverage significantly increased in both arms from preintervention to postintervention (OR 1.88, 95% CI 1.54 to 2.30; p<0.0005). In both health catchment areas, households whose CHW used UHC Mode had increased odds of minimum expected home visit coverage compared with households whose CHW used the control; however, the effect was only statistically significant in the larger health catchment area (Yirimadio: OR 2.40; 95% CI 1.66 to 3.49; p<0.0005 and Tori: OR=3.31; 95% CI 0.80 to 13.67; p=0.098) (table 3). We found no difference in the effect of UHC Mode by health catchment area (online supplemental appendix 3).

The effect of UHC Mode on minimum expected home visit coverage was significantly greater for households whose intervention CHW had the highest baseline service area coverage (>75th percentile) vs households whose intervention CHW had lower baseline service area coverage (25th to <75th percentile) (OR=3.28; 95% CI 1.30 to 8.26; p=0.012) (table 4 and online supplemental appendix 3).

**Table 4:** Exploratory analyses assessing differential effects of UHC Mode by baseline CHW service area coverage and household wealth (Tori only)

	Minimum expected home visit coverage		
	OR	95% CI	P value
Differential effects by CHW baseline service area coverage			
Treatment x post x CHW performance quartile			
<25th percentile	1.59	(0.73 to 3.43)	0.24
25th to <75th percentile (ref)	–	–	–
≥75th percentile	3.28	(1.30 to 8.26)	0.012
Differential effects by household wealth (Tori only)			
Treatment × post × household wealth quintile			
<20th percentile	0.46	(0.20 to 1.06)	0.069
20th to <40th percentile	0.47	(0.17 to 1.31)	0.15
40th to <60th percentile (ref)	–	–	–
60th to <80th percentile	0.39	(0.18 to 0.82)	0.013
≥80th percentile	0.43	(0.18 to 1.06)	0.067

Overall main effects model with the addition of a three-way interaction term.

CHW, community health worker; UHC, universal health coverage.

There were no differences in UHC Mode’s effect by actual household load (online supplemental appendix 4). In Tori, UHC Mode had a larger effect on minimum expected home visit coverage of households in the middle wealth quintile compared with those in the poorest and richest wealth quintiles, but estimated effects across quintiles were not significantly different from each other (table 4).

In sensitivity analyses, estimated effects of UHC Mode were robust to accounting for month fixed effects, alternative definitions of the preintervention period, and including zero home visit counts for inactive households (online supplemental appendix 2).

## Discussion

Use of UHC Mode more than doubled the odds of, and increased by 13 percentage points, minimum expected home visit coverage. Across two study areas, results were similarly positive, and suggest that UHC Mode was effectively designed for use across both types of settings.

Service area coverage of minimum expected home visits was consistently lower in the periurban versus the rural area during the study period. There are several possible explanations for this observation. First, due to rapid population growth and mixed migration dynamics in Bamako,^27^ maintaining accurate household registrations in the CHW application is more challenging for CHWs in the periurban area; this was the primary impetus that led to the development of the household muting form functionality. Although this functionality supports CHWs to maintain more accurate lists of active households in their service area, it still requires additional work to implement. Second, mean actual household load was higher in the periurban area vs the rural. Lastly, we suspect that family cohabitation structures in the rural area, where multiple related families reside together in a larger structural unit, were likely to be more conducive to conducting efficient home visits compared with the periurban area.

Although measures were taken to minimise potential contamination between study arms, which would bias results toward finding a null effect, positive spillover effects could have occurred between arms due to the nature of 360 Supervision^15^: UHC Mode and control CHWs shared supervisors, attended weekly group supervision sessions where they discussed common challenges and potential solutions, and received performance feedback via a CHW Supervision Dashboard featuring comparative metrics that may have promoted competition between CHWs. Although CHWs were informed during study recruitment that intervention assignment was not based on CHW performance, competition may still have influenced CHW behaviours, as previously reported.^15^

The temporal increase in minimum expected home visit coverage observed in both arms was likely due to software improvements made to both UHC Mode and control interventions by intervention launch, which included faster application performance and the ability for CHWs to temporarily or permanently inactivate households when they move from the service area. The household muting functionality was relevant given mixed migration in periurban Yirimadio and population movement in rural Tori, where violent conflict was an emerging issue.^27 28^

UHC Mode had different effects on minimum expected home visit coverage depending on CHW baseline service area coverage. UHC Mode’s effects were strongest for households whose CHW had the highest baseline service area coverage (>75th percentile). Although CHWs pilot testing UHC Mode were randomly selected, CHW selection and participation in focus groups and prototype development were biased toward the highest performers. Future product development should consider engaging more CHWs across the performance spectrum during prototype development and pilot testing. Lower-performing CHWs may also need more comprehensive support extending beyond a software tool. This underscores the importance of other critical programmatic strategies to address CHW performance disparities.

Previous programme records of the small area of Tori identified an important equity gap in minimum expected home visit coverage of households in the poorest wealth quintile, and we explored whether UHC Mode had any potential equity-enhancing effect. A subgroup analysis showed UHC Mode had similar effects in the poorest and richest household wealth quintiles, but this result was inconsistent across wealth quintiles and thus inconclusive. UHC Mode was not specifically designed to improve equity in minimum expected home visit coverage by household socioeconomic status. Further research and product development may test approaches to build on UHC Mode’s core design principles to identify and visually emphasise households considered more vulnerable within household lists.

Although this study’s results did not show that the effect of UHC Mode differed by actual household load, our analysis had limited ability to evaluate the impact of actual household load on minimum expected home visit coverage over time. The household muting functionality was implemented in both arms postintervention and CHWs required time to implement the feature. Measurement of actual household load over time was thus limited and subject to error. Our assumption that household loads were constant during the study period was flawed given mixed migration and population displacement that impacted study areas during the study period.^27 28^ The effect of UHC Mode is likely limited when the CHW-population ratio becomes unmanageable. Routine assessment and mapping of service areas to maintain optimal CHW-population ratios is important for preventing excessive workloads, which may result in decreased CHW motivation and performance.^29 30^

This study has certain limitations resulting from challenges in conducting operational research. The small sample size at Tori resulted in this site being underpowered. In addition, this study relied on CHWs to accurately and systematically record home visits, use the household muting functionality, and manage household registrations in the CHW application including registering new households in the service area. Although monthly audits of home visits were conducted by CHW supervisors (via in-person visits of randomly selected households as part of routine supervision) and study personnel (via telephone-based audits of randomly selected households to verify home visits and deactivations of households), audits were not systematically done for all CHWs. It is likely that calculations of service area coverage over time were subject to some measurement error. It was not possible to mask CHW participants and other programme personnel to arm allocation, which may have led to bias by supervisors and/or CHWs altering their behaviour. CHWs who used UHC Mode may have been less likely than CHWs who used the control to register new households, which would result in an overestimate of UHC Mode’s effect. The 4-month duration of the postintervention period was also relatively brief. Future research should evaluate whether UHC Mode can sustain improvements in minimum expected home visit coverage over a longer duration.

This study also has strengths that support its internal validity and potential generalisability. The nature of the intervention ensured CHWs adhered to their intervention assignment, and few CHWs were lost to follow-up. Positive results were observed across two study areas, one rural and one periurban, in different regions of the country. This study also featured a rigorous study methodology: a randomised design, which enhanced comparability between arms and minimised confounding and selection bias, and a difference-in-differences analysis, which controlled for any potential baseline differences between arms despite randomisation.

UHC Mode is a complement to, and not a substitute for, other important design elements of community health programmes, such as performance management and manageable CHW-population ratios.^14 16^ While our findings are contextually dependent on ProCCM, it is possible that UHC Mode may have similar positive effects in other areas of application requiring CHW follow-up, such as multiple-dose mass vaccination campaigns, prenatal care, and treatment of malnutrition or tuberculosis.

## Conclusion

As countries pursue their 2030 UHC goals, digitally supported CHWs can advance these goals at the local level. This trial is one of the first to test the effect of digital user interface design improvements on CHW performance, specifically minimum expected home visit coverage. Use of UHC Mode resulted in an increase in minimum expected home visit coverage. UHC Mode should be considered an effective tool that can improve minimum expected home visit coverage and promote progress towards UHC when implemented in the ProCCM context. Our positive results suggest that user interface design of health information systems that supports health workers in their daily practice and meets their requirements can have a positive impact on CHW performance and home visit coverage. In addition, the design and evaluation of community health information systems and tools that better support such operational tasks is a promising area of future research.

## Data Availability

Data are available on reasonable request. Investigators agree to share deidentified individual participant data, the study protocol, statistical analysis plan and analytical code (Stata) 6 months after publication and following the completion of a data use agreement. Proposals should be directed to info@musohealth.org.
