# Supplementary material for: Effect of mobile application user interface improvements on minimum expected home visit coverage by community health workers in Mali: a randomised controlled trial
Source: BMJ Glob Health. 2021 Nov 21;6(11):e007205. doi: 10.1136/bmjgh-2021-007205 (PMC8609935; doi:10.1136/bmjgh-2021-007205)
Supplement: Supplementary data [file bmjgh-2021-007205supp001.pdf]

Appendix to "Effect of mobile application user interface improvements on minimum expected home visit coverage by community health workers in Mali: a randomized controlled trial"

This appendix provides further methodological details for "Effect of mobile application user interface improvements on minimum expected home visit coverage by community health workers in Mali: a randomized controlled trial." This study appendix also provides a supplementary table showing the results of additional sensitivity analyses performed to check the robustness of our regression model, and exploratory analyses evaluating potential differential effects of the intervention.

Yang JE, *et al. BMJ Global Health* 2021; 6:e007205. doi: 10.1136/bmjgh-2021-007205

eAppendix 1. Data sources and preparation

Data sources
Prior to intervention launch, a baseline survey of participating CHWs' demographic characteristics was conducted to help assess randomization balance across study arms. Data on household wealth quintiles for households in Tori, acquired in a separate survey data collection exercise, were merged into this study's analytical data set.

Data preparation
To obtain comparable data for the duration of the study period, we aligned household definitions and data organization across Yirimadio and Tori health catchment areas in late January 2019. For Tori, this required modifying the data hierarchy in the standard CHW application, reorganizing concessions (structural units composed of multiple related families) into single families based on a household survey identifier number obtained from a separate survey data collection exercise which included a complete population census.[1] Among patients in the Bankass region with a valid census survey identifier number, 98.5% of patients were reorganized into single families.

eAppendix 2. Sensitivity analyses

Table S1. Sensitivity analyses of the difference-in-difference odds ratio for minimum expected home visit coverage from pre-intervention to post-intervention between arms

	Minimum expected home visit coverage		
	Odds Ratio	95% CI	p-value
Panel A: Inclusion of month fixed effects			
Treatment x Post	2.45	(1.69, 3.55)	<0.0005
Panel B: Alternative definitions of pre-intervention period			
Treatment x Post[1]	2.12	(1.48, 3.04)	<0.0005
Treatment x Post[2]	2.84	(1.91, 4.22)	<0.0005
Panel C: Inclusion of zero home visit counts for inactive households			
Treatment x Post[3]	2.07	(1.51, 2.84)	<0.0005

CI = confidence interval.
All model specifications feature estimates for a random effects panel regression, stratified by health catchment area, and a treatment indicator that takes value 0 for the control arm and value 1 for the intervention arm. All estimates are adjusted for clustering at the CHW level.
[1]Pre-intervention period defined as the four months with the CHW Supervision Dashboard (December 2018 - March 2019).
[2]With the month of UHC Mode roll-out (March 2019) coded as null.
[3]Including zero home visit counts for household months where households were indicated by CHWs to be temporarily or permanently inactive.

eAppendix 3. Exploratory analyses

Table S2. Exploratory analyses of differences in UHC Mode effects by health catchment area (Tori vs Yirimadio), levels of baseline service area coverage, and levels of CHW actual household load

	Minimum expected home visit coverage		
	Odds Ratio	95% CI	p-value
Differential effects by Health catchment area			
Treatment x Post x Health catchment area			
Yirimadio (ref)	–	–	–
Tori	1.32	(0.33, 5.19)	0.70
Differential effects by CHW baseline service area coverage			
Treatment x Post x CHW performance quartile			
<25th percentile (ref)	–	–	–
25th to <50th percentile	0.60	(0.25, 1.44)	0.26
50th to <75th percentile	0.69	(0.26, 1.83)	0.45
>=75th percentile	2.05	(0.80, 5.29)	0.14
Differential effects by Actual household load			
Treatment x Post x Actual household load quintile			
<20th percentile	0.72	(0.23, 2.27)	0.58
20th to <40th percentile	0.79	(0.25, 2.46)	0.69
40th to <60th percentile (ref)	–	–	–
60th to <80th percentile	0.88	(0.28, 2.73)	0.83
>=80th percentile	0.94	(0.31, 2.89)	0.92

CI = confidence interval.

Overall main effects model with the addition of a three-way interaction term.

Yang JE, *et al. BMJ Global Health* 2021; 6:e007205. doi: 10.1136/bmjgh-2021-007205
